# Improving Health Professional Recognition and Response to Child Maltreatment and Intimate Partner Violence: Protocol for Two Mixed Methods Pilot Randomized Controlled Trials

**DOI:** 10.2196/50864

**Published:** 2024-03-21

**Authors:** Melissa Kimber, Elizabeth Baker-Sullivan, Donna E Stewart, Meredith Vanstone

**Affiliations:** 1 Offord Centre for Child Studies Department of Psychiatry & Behavioural Neurosciences McMaster University Hamilton, ON Canada; 2 Department of Health Research Methods, Evidence, and Impact McMaster University Hamilton, ON Canada; 3 Department of Psychiatry University of Toronto Toronto, ON Canada; 4 Centre for Mental Health University Health Network Toronto, ON Canada; 5 Department of Family Medicine McMaster University Hamilton, ON Canada

**Keywords:** medical education, health professions education, child maltreatment, intimate partner violence, mixed methods, pilot trial, qualitative description, family violence

## Abstract

**Background:**

The optimal educational approach for preparing health professionals with the knowledge and skills to effectively recognize and respond to family violence, including child maltreatment and intimate partner violence, remains unclear. The Violence, Evidence, Guidance, and Action (VEGA) Family Violence Education Resources is a novel intervention that can be completed via self-directed learning or in a workshop format; both approaches focus on improving health professional preparedness to address family violence.

**Objective:**

Our studies aim to determine the acceptability and feasibility of conducting a randomized controlled trial to evaluate the effectiveness of the self-directed (experimental intervention) and workshop (active control) modalities of VEGA, as an adjunct to standard education, to improve learner (*Researching the Impact of Service provider Education [RISE] with Residents*) and independent practice (*RISE with Veterans*) health professional preparedness, knowledge, and skills related to recognizing family violence in their health care encounters.

**Methods:**

The *RISE with Residents* and *RISE with Veterans* research studies use embedded experimental mixed methods research designs. The quantitative strand for each study follows the principles of a pilot randomized controlled trial. For *RISE with Residents*, we aimed to recruit 80 postgraduate medical trainees; for *RISE with Veterans*, we intended to recruit 80 health professionals who work or have worked with Veterans (or their family members) of the Canadian military or the Royal Canadian Mounted Police in a direct service capacity. Participants complete quantitative assessments at baseline, after intervention, and at 3-month follow-up. A subset of participants from each arm also undergoes a qualitative semistructured interview with the aim of describing participants’ perceptions of the value and impact of each VEGA modality, as well as research burden. Scores on potential outcome measures will be mapped to excerpts of qualitative data via a mixed methods joint display to aid in the interpretation of findings.

**Results:**

We consented 71 individuals to participate in the *RISE with Residents* study. Data collection was completed on August 31, 2023, and data are currently being cleaned and prepared for analysis. As of January 15, 2024, we consented 34 individuals in the *RISE with Veterans* study; data collection will be completed in March 2024. For both studies, no data analysis had taken place at the time of manuscript submission. Results will be disseminated through peer-reviewed publications; academic conferences; and posting and sharing of study summaries and infographics on social media, the project website, and via professional network listserves.

**Conclusions:**

Reducing the impacts of family violence remains a pressing public health challenge. Both research studies will provide a valuable methodological contribution about the feasibility of trial methods in health professions education focused on family violence. They will also contribute to education science about the differences in the effectiveness of self-directed versus facilitator-led learning strategies.

**Trial Registration:**

ClinicalTrials.gov NCT05490121, https://clinicaltrials.gov/study/NCT05490121; ClinicalTrials.gov NCT05490004, https://clinicaltrials.gov/study/NCT05490004

**International Registered Report Identifier (IRRID):**

DERR1-10.2196/50864

## Introduction

### Background

The prevention of family violence, which includes intimate partner violence (IPV) and child maltreatment, remains a global public health priority. IPV encompasses a range of behaviors by a current or former romantic or sexual partner that causes or can cause physical, psychological, or sexual harm, including physical assault or violence, sexual coercion or assault, threats of harm, stalking or surveillance, and verbal degradation or humiliation [1]. Child maltreatment refers to adverse caregiver or parent behavior, including physical, sexual, or emotional abuse; physical or emotional neglect; coercive control; and exposure to IPV between adult caregivers, which result in actual or potential physical or emotional harm to the child. Globally, up to 1 in 3 children are exposed to at least 1 form of maltreatment before the age of 18 years [2-4]. Similarly, 27% of ever-partnered women between the ages of 15 and 29 years will experience IPV in their lifetime, with 24% of women aged 15-19 years and 26% of women aged 19-24 years having experienced IPV before the age of 15 years [5]. The literature details a significant and positive association between exposure to child maltreatment and increased vulnerability to IPV victimization over the life course [6,7]. In addition, a fulsome and consistent body of evidence demonstrates that exposure to child maltreatment or IPV is associated with a range of health-risk behaviors and negative health outcomes, including significantly elevated risk for early-onset smoking and alcohol use, teen pregnancy, underimmunization, obesity, heart disease, chronic pain conditions, substance abuse, suicide attempts, posttraumatic stress disorder, eating disorders, depression, anxiety, among others [1,8-10]. Importantly, the prevalence of child maltreatment and IPV in families of Active Duty and Veteran service members is even higher. For example, nearly 63% of Active Duty and Veteran members of the Canadian Armed Forces (CAF) report exposure to maltreatment in childhood [11], and upward of 25% of Active Duty or Veteran members of the CAF (or whose partner is a member or a Veteran of the CAF) self-report IPV victimization or perpetration in their lifetime [12-14]. Among this subgroup of the population, a history of child maltreatment or IPV exposure is also associated with a range of physical and mental health disorders, which can be exacerbated by deployment-related traumatic experiences, including military sexual trauma; receipt of incoming artillery, rocket, or mortar fire; or knowing someone seriously injured or killed while deployed [11,15].

Critically, a growing compilation of clinical guidance and guidelines indicates that the probability for negative health outcomes related to family violence exposure, including child maltreatment and IPV, can be attenuated via early interaction with a health care professional that is considerate and who prioritizes not only physical and emotional safety in the health care encounter but also the broader social, psychological, and physical health needs of an exposed patient [4,16-20]. For this reason, health professionals have been identified as having an essential role in family violence prevention via recognizing and responding to this exposure and its associated sequelae in their health care practice. Unfortunately, several studies detail uniform challenges to family violence recognition and response across the health professions, including limited formal curriculum during preservice training, discomfort and a lack of confidence related to asking about and responding to family violence disclosures, and the perception that there is insufficient time to adequately address family violence disclosures in health care practice [21]. Paralleling these barriers is a dearth of evidence regarding how to best prepare health professionals with the knowledge and skills to effectively recognize and respond to suspected or disclosed family violence exposures in health care contexts [21,22]. It is also unclear whether family violence education efforts should be optimally targeted to preservice versus in-service health professionals, nor is it clear the extent to which repeated exposure to family violence curriculum is necessary to achieve and maintain professional competencies in this area.

Broadly, educational interventions focusing on family violence, which have been evaluated in undergraduate, postgraduate, and continuing education contexts, vary in their instructional approaches and often fail to consider active controls in their research designs [22-27]. In addition, there has been limited emphasis on developing and evaluating interventions that address the complex overlap between IPV, children’s exposure to IPV, and other forms of child maltreatment [28,29]. Given the prevalence, overlap, and health-related burdens of child maltreatment, IPV, and children’s exposure to IPV, there is an urgent need to identify empirically supported educational interventions that adequately prepare the health professionals to care for individuals and families impacted by all forms of family violence. Additionally, the field of health professions education stands to benefit from research studies that provide evidence for the optimal educational approach (eg, self-directed learning vs facilitator-based approaches) to improve health professionals’ knowledge and skills in complex areas, such as family violence. This methodological contribution can also offer guidance on whether the optimal educational approach varies according to the health professional’s status as a learner or an independent practitioner.

### Aims

The objective of our 2 complementary studies are to determine the acceptability and feasibility of conducting a randomized controlled trial (RCT) to evaluate the effectiveness of the self-directed (experimental intervention) and workshop (active control) modalities of the Violence, Evidence, Guidance, and Action (VEGA) Family Violence Education Resources [30], as an adjunct to standard education, to improve learner (*Researching the Impact of Service provider Education [RISE] with Residents*) and independent practice (*RISE with Veterans*) health professional preparedness, knowledge, and skills related to recognizing family violence in their health care encounters. Detailed information about the VEGA Family Violence Education Resources (hereafter referred to as “VEGA”) is available on the web [31] and in the *Methods* section. Briefly, VEGA was developed based on systematic reviews and consultations with members of 22 national health care and social service organizations in Canada [30]. VEGA is a web-based suite of resources that uses a participatory, encounter-focused curriculum across 4 learning modules that focus on the following areas of family violence: (1) the epidemiology of child maltreatment, IPV, and children’s exposure to IPV in Canada; (2) evidence-informed strategies for safely recognizing and responding to (a) child maltreatment and (b) IPV (including children's exposure to IPV) in health care encounters; and (3) principles for ensuring physical and emotionally safe health care encounters for family violence discussions [30]. VEGA can be completed as a self-directed educational activity (ie, self-directed VEGA) via completion of the web-based modules at the learner’s own pace, or as a remote or face-to-face workshop (ie, workshop VEGA). VEGA workshops are delivered by trained facilitators who are also regulated and practicing health care professionals; both self-directed VEGA and workshop VEGA take approximately 3 hours to complete [21,30].

## Methods

### Study Design

The *RISE with Residents* and *RISE with Veterans* research studies use quantitatively dominant, embedded experimental mixed methods research design (“QUAN(qual)”) [32]. The use of this design allows for the measurement of important acceptability and feasibility metrics related to enrollment, retention, attrition, and data completeness and the generation of exploratory estimates of the education effect for both VEGA modalities. This design also allows for the systematic collection of qualitative data to provide important contextual information regarding the tenability of a full RCT, as well as a description of how VEGA modalities influence any measured changes in health professional preparedness, knowledge, and skills related to recognizing and responding to family violence. Given the cost and complexity of implementing RCTs, as well as well-documented challenges related to recruiting and retaining health professionals in clinical and education research [33-35], pilot randomized studies that allow for responsive amendments to recruitment, retention, and data collection are an imperative proviso to reduce the possibility of failed or incomplete phase-3 RCTs [36-38].

### Quantitative Strand of Data Collection

#### Quantitative Design and Participants

**Figure 1:**
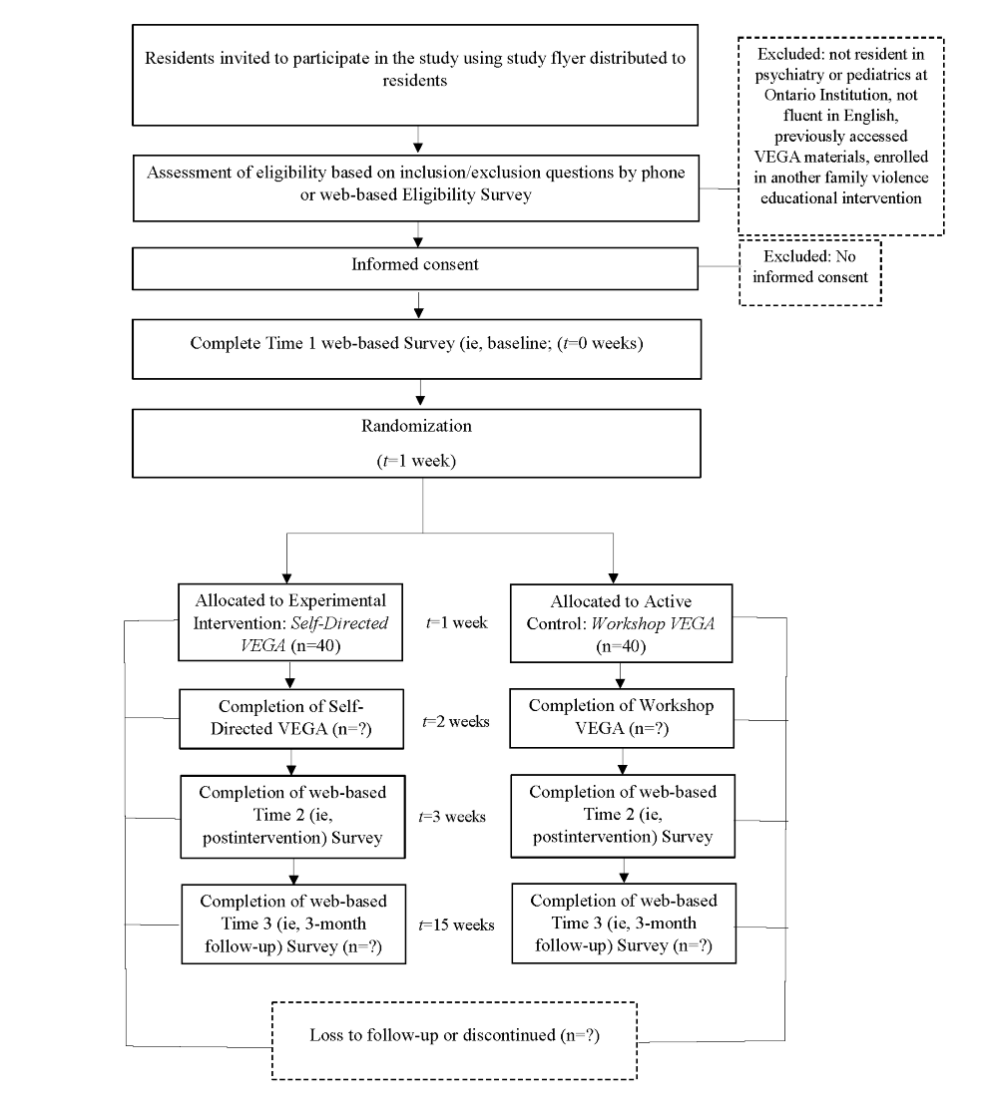
RISE with Residents SPIRIT flow diagram. RISE: Researching the Impact of Service provider Education; SPIRIT: Standard Protocol Items: Recommendations for Interventional Trials; VEGA: Violence, Evidence, Guidance, and Action.

**Figure 2:**
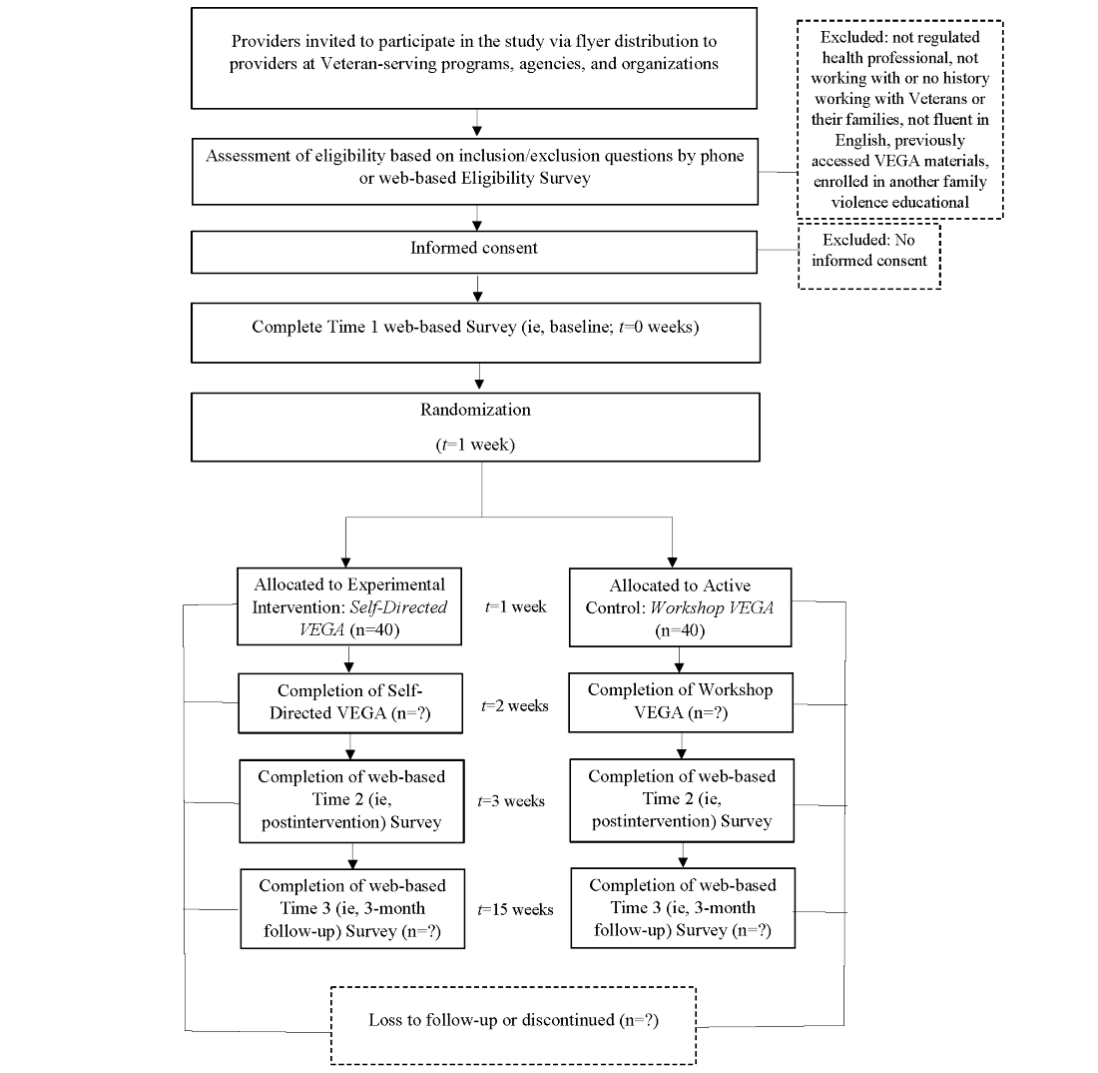
RISE with Veterans SPIRIT flow diagram. RISE: Researching the Impact of Service provider Education; SPIRIT: Standard Protocol Items: Recommendations for Interventional Trials; VEGA: Violence, Evidence, Guidance, Action.

The quantitative research design for both studies follows the principles of a 2-armed pilot RCT [37,38]. Figures 1 and 2 detail the SPIRIT (Standard Protocol Items: Recommendations for Interventional Trials) flow diagrams for *RISE with Residents* and *RISE with Veterans*, respectively [39,40]. For *RISE with Residents*, we aimed to recruit a voluntary sample of 80 participants from postgraduate medical residency programs in psychiatry or pediatrics in Ontario, Canada. Directors of each program were asked to circulate recruitment-related materials to their respective program residents, requesting that interested individuals contact the research team to determine eligibility and complete consenting procedures. For *RISE with Veterans*, we aimed to recruit a voluntary sample of 80 health professionals who currently work or have worked in a direct service capacity with Veterans of the Canadian military or the Royal Canadian Mounted Police (RCMP), or their family members, to participate. Directors of Veteran-serving programs, agencies, and organizations had the opportunity to meet with members of the research team to discuss the components of the study and were asked to distribute recruitment materials to potentially eligible staff. Recruitment materials requested that those who were interested in participating contact the research team.

#### Eligibility Criteria

Eligibility screening was completed over the phone with the study research coordinator (RC) or electronically via REDCap (Research Electronic Data Capture; Vanderbilt University) using the eligibility criteria outlined in Textbox 1. At this time, we also collected sociodemographic characteristics of participants, including age, sex at birth, and self-identified gender. For *RISE with Residents*, we also included information about their residency program; *RISE with Veterans* additionally asked questions about the organization the participants work for, whether they work directly with Veterans of the military or the RCMP, or their family members, and if the latter, whether consent is required by the Veteran for the participant to provide services to the Veteran’s family member. We obtained consent from potential participants to keep their responses to the screening measures in cases they were ineligible or did not end up participating in the study. This was for the purpose of comparing those who end up participating with those who do not end up participating to understand if our criteria are systematically excluding any groups. After completing screening, individuals were informed of their eligibility to participate and underwent an electronic informed consent process to participate in the remainder of research activities. Potential participants were provided the option to speak with a member of the research team to review any questions about the consent procedures and research process.

**Eligibility criteria.**

**Inclusion criteria**

- *Researching the Impact of Service provider Education (RISE) with Residents*
  Physician resident enrolled in a postgraduate medical residency program in psychiatry or pediatrics within Ontario, Canada
  Willing and able to provide informed written consent and complete all project activities in English
- *RISE with Veterans*
  18 years of age or older
  Regulated health care or social service professional
  Working with military or Royal Canadian Mounted Police (RCMP) Veterans, or military or RCMP Veteran’s family members in a direct service capacity at least 1 day per week
    OR
    Have 2 years of experience working with military or RCMP Veterans, or their family members, in a direct service capacity
    OR
    Have worked with 15 or more clients or patients who were either military or RCMP Veterans, or their family members, in a direct service capacity
  - Willing and able to provide informed written consent and complete all project activities in English

**Exclusion criteria**

- *RISE with Residents*
  Have previously accessed the Violence, Evidence, Guidance, and Action (VEGA) web-based or workshop materials
  Are currently enrolled in or expected to enroll in any other educational intervention focused on family violence (intimate partner violence [IPV], child maltreatment, childhood exposure to IPV) within the study time period
- *RISE with Veterans*
  Have previously accessed the VEGA materials
  Are currently enrolled in or expected to enroll in any other educational intervention focused on family violence (IPV, child maltreatment, or childhood exposure to IPV) within the study time period

#### Randomization and Concealment

Randomization occurred after consent has been obtained and the web-based baseline survey (ie, time 1 survey, see below) is complete. To reduce the possibility of “availability bias,” we required the accumulation of 20 consenting participants who indicated their availability to attend a VEGA workshop before randomization, as each VEGA workshop requires a minimum of 10 participants. The cohort of 20 consenting participants were then randomly assigned to the experimental (ie, self-directed VEGA) or active control (ie, workshop VEGA) condition using stratified block randomization [41] with a block size of 4 (blocking factor of 2), using a third-party, internet-based randomization service, Randomize.net. For *RISE with Residents*, randomization was stratified based on two variables: (1) sex at birth (female, male, intersex, or prefer not to answer) and (2) discipline (pediatrics vs psychiatry). For *RISE with Veterans*, randomization was stratified based on sex at birth only. Sex at birth, as opposed to gender identity, was selected as the stratification variable because of a limited number of categories needed to achieve balance across trial arms. The analysis section outlines our proposed approach to disaggregate sex- and gender-based data after the conclusion of both studies.

#### Masking

Given the nature of experimental and control arms, it is impossible to conceal allocation status from participants, as well as the facilitators of the active control arm (ie, workshop VEGA). Participants were informed of their allocation status by the RC. The RC did not reveal participant allocation status to the study research assistant, the latter of whom was responsible for ensuring quantitative data collection. Participants were also encouraged not to inform the research assistant of their allocation status.

#### Intervention

##### VEGA Family Violence Education Resources

Time for completion of self-directed VEGA or workshop VEGA is approximately 3 hours, and pedagogical elements for both approaches have been informed by education scholarship (see Table 1) [42-51]. Participants had or have the option of completing self-directed VEGA in either English or French as the VEGA website offers the content in both languages. Workshop VEGA is delivered in English only in a remote or in-person workshop format by trained facilitators who are regulated health professionals. VEGA workshops are recommended to have a 10:1 participant-to-facilitator ratio and are standardized via the use of a flexibly structured facilitator guide [30].

**Table 1:** Pedagogical elements of self-directed VEGA (ie, experimental arm) and workshop VEGA (ie, active control arm) educational approaches.

Pedagogical elements	Self-directed VEGA^a^ (experimental arm)	Workshop VEGA (active control arm)
Didactic material [48]	Asynchronous reading	Synchronous lecturing with 2 facilitators
Deliberate practice [49,50]	Case-based animated simulations	Case-based role play
Enabling learning tools [51]	Remote patients, clinical handbook, clinical scripts	Remote patients, clinical handbook, clinical scripts
Test-enhanced learning [46]	Individual multiple-choice questions with response feedback	Group-based polling (ie, multiple-choice) and feedback

^a^VEGA: Violence, Evidence, Guidance, and Action.

##### Experimental Arm (Self-Directed VEGA)

If a participant is randomized to the experimental arm, they will be or were asked to complete self-directed VEGA at their convenience, within 1 week of being informed of their allocation status. To complete the intervention, individuals are asked to register their access to the VEGA Resources and review and complete all module activities at their own pace during the weeklong intervention period [30].

##### Active Control Arm (Workshop VEGA)

If a participant is randomized to the active control arm, they will be or were informed that they need to attend a remote VEGA workshop. Because of COVID-19 restrictions in place at the outset of both studies, VEGA workshops take place via Zoom and are delivered by 2 trained facilitators. Workshops include 10 to 20 participants, keeping the recommended 10:1 participant-to-facilitator ratio, and last 3 hours [30].

##### Intervention Adherence

Intervention adherence is monitored by the RC. The RC has received or will receive attendance reports for participants allocated to VEGA workshops from the VEGA project team, who will also provide the research team confirmation of whether self-directed participants completed the “Self-Declaration of Module Completion Form” embedded within the self-directed VEGA website. Self-directed VEGA participants receive email reminders from the RC every 2 days to complete the modules within the intervention window [30].

##### Data Collection

The primary outcomes for the *RISE with Residents* and *RISE with Veterans* research studies are related to the acceptability and feasibility of RCT implementation. To this end, the RC tracked or is tracking the number of individuals for each study who (1) agreed to be screened, (2) are eligible for participation, and (3) enroll. We are also recording the number of (4) emails or phone calls needed to arrange all research assessments and the number of participants who (5) drop out, (6) could not be reached for follow-up, (7) complete the interventions, and (8) partially complete versus fully complete secondary outcome research assessments. As an indicator of feasibility, we are also recording the total time to complete all secondary outcome research assessments and track the number of protocol amendments (if any) and their rationale. Acceptability and feasibility metrics will be matrixed alongside sociodemographic characteristics (sex at birth, gender, age, etc) of screened and consented participants.

Table 2 details the list the measures that are being used to assess the secondary (ie, education-related) outcomes and their correlates in the *RISE with Residents* and *RISE with Veterans* research projects and their respective overlap. The research assistant is administering research assessments of family violence knowledge and skills via REDCap to participants in both studies at 3 time points: the week before they begin their VEGA intervention (time 1), posteducation (ie, postintervention; time 2), and 3 months after the time 1 survey is completed (time 3). Participants are being asked to complete the surveys within 1 week, from the date they are initially sent, on their own time. Each assessment takes approximately 30 minutes to complete.

**Table 2:** Quantitative research assessments for *RISE with Residents* and *RISE with Veterans*.

Measure	*RISE^a^ with Residents*			*RISE with Veterans*		
	Baseline	Posteducation	3-month follow-up	Baseline	Posteducation	3-month follow-up
Adapted Brief Individual Readiness to Change Scale (BIRCS) [52]: The original BIRCS was developed by Goldman [52] to assess health professional readiness to learn and implement new evidence-based practices in the field of addictions; respondents were asked to indicate on a 5-point Likert scale the extent to which they disagreed (0=strongly disagree) or agreed (4=strongly agree) with 5 statements about their use of “direct service techniques that are based on research” (eg, I believe I have the skills to use them; I believe I have the flexibility to use them). For the purposes of our research, we adapted the items to be specific to child maltreatment, added 2 items (eg, “I believe I have the knowledge to recognize and respond to all forms of child maltreatment in my practice”; “I am motivated to learn about child maltreatment”), and replicated the set of items for intimate partner violence (IPV) (ie, BIRCS—Child Maltreatment; BIRCS—IPV). After reverse scoring 1 item, the mean score of the responses for each scale is used to interpret the results, with a higher mean score indicative of a greater readiness to make practice changes related to child maltreatment or IPV.	✓	_—b_	—	✓	—	—
Adapted Child Maltreatment Vignette Scale (CMVS) [53,54]: For the original CMVS, respondents are prompted to review 14 distinct analog vignettes that depict a range of signs and symptoms of child maltreatment exposure. On completing their review of each vignette, participants are asked to indicate their responses to four items: (1) “is this child being maltreated” (yes/no); (2) “please indicate how confident you are in your response (50%-100%, your answer must be between 50% and 100%)”; (3) “would you report this case to children’s services?” (yes/no); and (4) “please indicate how confident you are in your response (50%-100%, your answer must be between 50% and 100%).” With permission from the original authors, the measure was adapted to the Canadian context via removing 1 scenario from the measure (due to concerns about multiple maltreated children), amending physician-focused language to “health professional” and changing (1) for each vignette to “for any child/youth in this scenario, do you have a reason to suspect child maltreatment?” (yes/no) and changing (3) for each vignette to “would you report this case to Child Welfare Services?” (yes/no). Adaptations were made in consultation with clinical experts in child maltreatment impact assessment at a tertiary care center in Ontario, Canada. Responses to (1) and (3) for each vignette will be scored as either correct (“1”) or incorrect (“0”) as predetermined, a priori; a mean “knowledge and skill accuracy” score is generated for analysis, with higher scores indicative of greater knowledge and skill accuracy related to child maltreatment.	✓	✓	✓	✓	✓	✓
Mandatory Reporting Self-Efficacy Scale (MRSES) [55]: The MRSES is a 7-item measure that asks respondents to indicate the extent to which they perceive their ability to implement a series of behaviors related to mandatory reporting of child maltreatment. Response options for each item are anchored on a scale from 0 to a 100, with statements at 0, 50, and 100 indicating “cannot do at all (0),” “moderately can do (50),” and “highly certain can do (100).” A total score is generated by summing items across the scale for each participant, with higher scores indicative of greater self-efficacy related to recognizing and reporting suspected child maltreatment.	✓	✓	✓	✓	✓	✓
Adapted Preparedness Subscale of the Physician Readiness to Manage Intimate Partner Violence Survey (PREMIS) [56,57]: The PREMIS is a 67-item self-report tool that was developed to assess physician management of IPV across 10 subscales. The Preparedness Subscale asks respondents to indicate the extent to which they feel prepared to address various aspects of IPV recognition and response when working with their clients across 11 items; these aspects include the conduct of safety assessments, asking appropriate questions about IPV, responding to IPV disclosures, among others. Response options are on a 7-point Likert scale ranging from “not prepared” (1) to “quite well prepared” (7), and items are averaged to generate a mean score for practitioner preparedness, with higher scores indicative of generally greater preparedness to recognize and respond to IPV. Preparedness items were adapted to focus on child maltreatment, allowing our team to determine participant preparedness to recognize and respond to both IPV and child maltreatment in their practice encounters.	✓	—	✓	✓	✓	✓
Adapted Knowledge Subscale of the Physician Readiness to Manage Intimate Partner Violence Survey (PREMIS) [56,57]: The knowledge subscale of the PREMIS assesses the accuracy of participants actual knowledge about IPV against a set of multiple-choice, matching, and true-or-false questions capturing information about IPV signs and symptoms and risk factors that are informed by the current literature. A total score of correct items is used to represent actual IPV knowledge, with higher scores indicative of greater knowledge.	—^b^	—	—	✓	✓	✓
Adapted Opinions and Self-Efficacy Subscale of the Physician Readiness to Manage Intimate Partner Violence Survey (PREMIS) [56,57]. The opinions (24 items) and self-efficacy (3 items) subscale of the PREMIS assesses participants’ agreement with statements capturing thoughts and beliefs related to recognizing and managing IPV in clinical practice. Response options are on a 7-point Likert scale ranging from “strongly disagree” (1) to “strongly agree” (7); items are averaged to generate a mean score for opinions and self-efficacy, with higher scores indicative of generally positive opinions and self-efficacy to recognize and manage IPV in practice. Developed in the United States, the original scale contained an item related to state-specific reporting of “IPV, elder abuse, and child abuse”; this item was removed for our studies to reduce redundancy with other measures and to reduce measurement burden. Self-efficacy items were asked at all time points in the study, with opinions items asked at baseline and 3-month follow-up.	—	—	—	✓	✓^c^	✓
Thoughts and Beliefs about Role Responsibility to Recognize and Respond to Family Violence (TBR-FV) [21]. The TBR-FV is a measure created by our research team that captures participants’ perceived professional responsibility related to recognizing and responding to IPV and child maltreatment in their health professional encounters. The generation of this measure was informed by a mixed methods program of research evaluating health professions education in family violence [21].	✓	—	—	✓	—	—
Healthcare Provider Attitudes toward Child Maltreatment Reporting Scale (HPA-CMRS) [58,59]: The HPA-CMRS is a 26-item psychometrically validated scale that asks respondents to indicate the extent to which they agree with statements that capture attitudes and beliefs regarding the reporting of child maltreatment to child protective services. Items are rated on a 5-point Likert scale ranging from “0” (strongly disagree) to “4” (strongly agree) and summed to produce a total score, with higher total scores indicative of more positive attitudes and beliefs toward the reporting of child maltreatment.	✓	—	✓	—	—	—
Adapted Version of the Achievement Goals for Work Domain (AGWD) [60,61]: The AGWD is a 23-item, psychometrically validated measure of work-related achievement goals that map onto the 4 goal orientations described by Achievement Goal Theory. Respondents are asked to indicate their agreement with 22 statements that follow the stem of “In residency, my goal is...” Response options range from “1” (strongly disagree) to “7” (strongly agree), and responses are summed to generate a total score for each subscale corresponding to each type of goal orientation; higher scores are more indicative of the respondent’s affinity to that goal orientation.	✓	—	—	—	—	—
Sociodemographics (current age, sex at birth, current gender identity, race, child maltreatment training history, IPV training history, etc)	✓	—	—	✓	—	—

^a^RISE: Researching the Impact of Service provider Education.

^b^Not applicable.

^c^Only the self-efficacy items will be administered at this time point.

#### Sample Size

A sample size of 80 participants (40 per arm) for each study was selected based on the recommendations of Whitehead et al [62] and Norman et al [63]. Based on these guidelines, a sample of 40 participants randomly allocated to each intervention arm would provide, at minimum, 80% power to detect a moderate (0.5 SD to 0.6 SD) effect size, which is indicative of a clinically significant change. In following this algorithm, our team will be able to consider the needed recruitment and retention rates to successfully implement a definitive education trial.

### Qualitative Strand of Data Collection

#### Qualitative Design and Participants

The qualitative portion of both studies is guided by the principles of qualitative description, which is a flexible yet rigorous approach to conducting qualitative health research that has clinical and practical relevance [64]. Qualitative description is being used to expand and extend what we learn about acceptability and feasibility of implementing the experimental and active control interventions and associated research activities, as well as capture the perceived value and impact of VEGA’s educational modalities, using the language of participants. Given that the qualitative data will complement the quantitative strand of data collection, we used purposive criterion sampling [65] to select a subsample of the participants from the active control and experimental arms of each study (n=60; 15 per arm, per study) to participate in a one-on-one semistructured interview with an unmasked member of the research team. For *RISE with Residents*, recruitment of qualitative participants is stratified by resident discipline and residency year, whereas for *RISE with Veterans*, qualitative recruitment is stratified by participants’ sex at birth (male vs female).

#### Data Collection

Individual semistructured interviews take place after intervention completion. Interviews are up to 60 minutes in length and conducted via Zoom using the audio function only (or by phone, if the participant prefers). Interviews are being audio-recorded and transcribed verbatim to ensure data integrity. A semistructured interview guide of 5 to 7 key questions and probes is used to guide interview experiences. Field notes completed by the interviewer document interview observations that may be relevant to analysis.

### Quantitative and Qualitative Data Analysis and Integration

On the completion of data cleaning, descriptive statistics (means, SDs, relevant quantiles, and proportions) will be used to compare VEGA modalities with respect to measures taken at baseline to ensure that groups do not significantly differ on sociodemographic characteristics [66]. The proportion of participants who met eligibility requirements, who enrolled, who were lost to follow-up, and who completed all quantitative secondary outcome assessments will be calculated. Assuming a normal distribution, standardized effect estimates for each of our educational outcomes in the form of Cohen *d* will be calculated for postintervention and 3-month follow-up time points. These data will be analyzed using intention-to-treat analysis. Depending on acceptability and feasibility outcomes, sample size calculations for a proposed RCT will be generated using the effect size and variance estimates from the posteducation change data for the selected outcome measures. Although forced-choice frameworks in REDCap reduce the proportion of missing data at the item level, missing follow-up data will be addressed using imputation procedures, where appropriate [67].

Transcripts of qualitative interviews, as well as associated field notes, are being exported into and managed in NVivo (Lumivero). On completion of both studies, transcripts will be analyzed using reflexive thematic analysis [68] and the constant comparison technique, which will allow for the identification codes, categories, and themes related to implementing and evaluating both VEGA modalities among health professionals in a postgraduate training (ie, learner) and independent practice setting. After conducting separate quantitative and qualitative analyses, quantitative and qualitative data will be integrated for interpretation via a mixed methods joint display [69]. Quantitative acceptability and feasibility metrics will be mapped to excerpts of qualitative data on perceived acceptability or educational burden; this joint display will support a comprehensive interpretation of the extent to which definitive trials examining VEGA in our sample populations are tenable. A separate joint display will cross-tabulate scores on secondary outcome measures with qualitative excerpts of VEGA’s perceived value and impact for improving health professional knowledge and skills in family violence.

### Ethical Considerations, Protocol Deviations and Amendments

Risks associated with the *RISE with Residents* and *RISE with Veterans* studies are minimal. All de-identified data are being stored on a secure server at McMaster University, as approved by the Hamilton Integrated Research Ethics Board (HiREB). Any identifying data will be destroyed on completion of both studies. Only the research team has access to study-related data; anyone outside of the research team who wishes to analyze the data can request to do so via formal secondary data analysis approval procedures administered via the HiREB. Anticipated adverse events that are relevant to this study include those related to participant safety and well-being and include (1) participants’ own experiences with IPV or child maltreatment, which may raise or contribute to distress during the educational intervention or during research activities, and (2) participants’ experiences with providing care to patients who have experienced IPV or child maltreatment, which may be distressing. Anticipated adverse events that are not serious are discussed, as needed by research staff, with the principal investigator if the nature of the adverse event is considered to signal unresolved risk to the participant. VEGA workshop facilitators are regulated health professionals with significant training and expertise in distress protocols. As per our protocols, emergency medical services are alerted as required if there is concern about imminent risk to life of an adult or safety of a child. The principal investigator, who is a registered social worker and psychotherapist, continues to follow regulated reporting requirements as necessary and determine whether other steps are needed to mitigate any risks to participants. Given that the purposes of both studies are to determine acceptability and feasibility of RCT implementation, a detailed accounting of any adverse events, research protocol deviations, and amendments continues to be tracked and documented by the RC, in collaboration with the principal investigator. Given the emphasis of both studies on the acceptability and feasibility of proposed RCT procedures, a data monitoring committee has not been established; however, findings from the present studies will inform the development of a data monitoring committee should results indicate that pursuit of a full RCT is acceptable and feasible.

### Ethics Review

The *RISE with Residents* and *RISE with Veterans* research projects have been approved for human research by the HiREB, affiliated with McMaster University in Ontario, Canada. The associated project approvals are HiREB #14381 (*RISE with Residents*) and HiREB #14243 (*RISE with Veterans*), respectively. All research procedures will be performed in accordance with the relevant guidelines and regulations of the HiREB and Tri Council Policy Statement on the Ethical Conduct for Research Involving Humans. In both studies, informed consent for study participation is obtained from all participants.

## Results

### Study Timeline

The *RISE with Residents* and *RISE with Veterans* studies were registered and posted to ClinicalTrials.gov on August 5, 2022. As of January 15, 2024, we consented 71 individuals to participate in the *RISE with Residents* study and data are currently being cleaned and prepared for analysis. A total of 6 amendments were submitted to the HiREB throughout the duration of the *RISE with Residents* data collection period; no adverse events were reported by research participants. For the *RISE with Veterans* study, as of January 15, 2024, we have consented 34 individuals to participate in the study and data collection will be completed in March 2024. At the time of writing (January 2024), 7 amendments were submitted to the HiREB and no adverse events have been reported by research participants.

### Dissemination Plan

In addition to the open-access publication of our research protocol, our team has identified several strategies that will accelerate the translation of our findings into education guidelines, practice, and scholarship. First, we will publish study findings for both the *RISE with Residents* and *RISE with Veterans* studies in open-access, peer-reviewed journals according to reporting guidelines for mixed methods [70], pilot trial [71], and education studies [72]. Second, end-of-grant knowledge translation will involve the preparation of 1-page infographics and curriculum recommendations for project funders at the Royal College of Physicians and Surgeons of Canada, the Atlas Institute for Veterans and their Families, and our network of health professions associations; representatives from each association generously disseminate our knowledge translation products via posts to their website, social media channels, and listserve platforms. Finally, we will present our findings at national and international conferences focused on health professions education and education scholarship.

## Discussion

### Key Findings

The *RISE with Residents* and *RISE with Veterans* research studies aim to determine the acceptability and feasibility of conducting an RCT to evaluate the effectiveness of the self-directed (experimental intervention) and workshop (active control) modalities of the VEGA Family Violence Education Resources [30], as an adjunct to standard education, to improve learner (*RISE with Residents*) and independent practice (*RISE with Veterans*) health professional preparedness, knowledge, and skills related to recognizing family violence in their health care encounters. We compare these 2 educational modalities for 2 key reasons. First, evidence indicates that self-directed education may be as effective as traditional education methods for improving knowledge and skills among health professionals, especially when self-directed methods incorporate active learning strategies [73,74]. Given that VEGA is a free, online, and brief intervention, determining the extent to which self-directed education yields improved preparedness, knowledge, and skills among learner and independently practicing health professionals, as an adjunct to formal curriculum, could (1) widely (and rapidly) shift the preparation of Canadian health professionals to effectively recognize and respond to family violence without requiring formal curricular changes and (2) meaningfully contribute to our understanding about effective postgraduate and continuing education strategies, which has education implications beyond family violence and the VEGA intervention.

Second, the comparison of educational modalities in our respective studies minimizes the variability that could not be accounted for if VEGA were compared with a non-VEGA control; this includes variability related to intervention content and emphasis, student enthusiasm, among others. RCTs in education scholarship have been controversial because they typically average too many variables to yield any real insights [75,76]. This is particularly true when an intervention is compared with a passive control or an active control that is focused on a separate subject. By keeping the content standard, varying only the educational modality, and placing emphasis on evaluating the acceptability and feasibility of an RCT, both studies address each of these important issues [77-79].

### Strengths and Limitations

A particular strength of the *RISE with Residents* and *RISE with Veterans* research studies lies in their emphasis on postgraduate and continuing education contexts. It is possible that family violence education may be especially impactful in the postgraduate training period given the need to (1) understand that children, youths, and adults can present with signs and symptoms of all types of family violence in every health professional subspecialty and (2) demonstrate competencies related to the scope of one’s professional responsibilities (eg, mandatory reporting) and the purview of one’s clinical practice, including when and how to make appropriate treatment referrals to prevent or reduce mental or physical health impairment. To this end, the results of the *RISE with Residents* and *RISE with Veterans* studies provide a valuable methodological contribution about the feasibility and acceptability of trial methods in postgraduate and continuing education focused on family violence in the Canadian context; it will also contribute new knowledge to education science about the differences between the effectiveness of self-directed versus facilitator-led learning strategies at different stages of the learning trajectory, more broadly.

The limitations of the *RISE with Residents* and *RISE with Veterans* research studies are influenced by the scope of the available literature on effective interventions for preventing family violence and mitigating associated harms, more generally. For example, it is important to note that IPV occurs in all countries, cultures, religions, and socioeconomic groups in the world. However, generally speaking, IPV is a gendered phenomenon; cisgender and transgender women and girls are disproportionately affected by IPV. Yet, increasing evidence indicates that IPV may be perpetrated by men toward women and by women toward men and occur in same-sex, gender, and sexually diverse relationships [10]. IPV may materialize in marital relationships, common-law relationships, cohabitation, or any intimate relationship including dating and casual sexual relationships across the age spectrum [10]. Yet, most epidemiological and intervention data related to IPV and its prevention have been collected in the context of cisgender, heterosexual adult relationships; thus, the information contained in VEGA is largely informed by that lens. Similarly, information regarding evidence-based approaches, practices, and programs for individuals who use violence (ie, perpetrators of IPV or child maltreatment) is limited. This limitation is also reflected in the evidence reviews and available guidance for working with perpetrators contained within the VEGA education modalities [10,80-82]. Finally, and methodologically, our quantitative measures rely on learner and independent practitioner self-report, which can be prone to bias. However, our selected measures have undergone psychometric scrutiny and heavily rely on a behavioral intention framework, which is consistent with the concept of self-efficacy (ie, beliefs about capabilities)—a construct with moderate-to-strong associations with provider behavior change [83,84].

### Conclusions

We expect that the *RISE with Residents* and *RISE with Veterans* research studies will provide critical evidence related to the acceptability and tenability of evaluating VEGA in postgraduate and continuing education settings. Both studies will also provide foundational estimates of intervention impact among 2 distinct populations. To this end, the findings have broader implications for the possibility of improving the preparation of health professionals to be able to recognize and respond to family violence in their care encounters safely and effectively. The generated effect estimates will serve as benchmarks for replication and, more specifically, the design of adequately powered and methodologically robust evaluations of the VEGA and other family-violence focused educational interventions over the long term.

## Abbreviations

CAF: Canadian Armed Forces
HiREB: Hamilton Integrated Research Ethics Board
IPV: intimate partner violence
RC: research coordinator
RCMP: Royal Canadian Mounted Police
RCT: randomized controlled trial
REDCap: Research Electronic Data Capture
RISE: Researching the Impact of Service provider Education
SPIRIT: Standard Protocol Items: Recommendations for Interventional Trials
VEGA: Violence, Evidence, Guidance, and Action

## Data Availability

To protect human privacy, data are not publicly available. Should researchers wish to complete secondary analysis of the *RISE with Residents* and *RISE with Veterans* research data, they may formally apply with a request to do so via the Hamilton Integrated Research Ethics Board (HiREB).
